# Evaluation of Recombinant Multi-Epitope Outer Membrane Protein-Based *Klebsiella pneumoniae* Subunit Vaccine in Mouse Model

**DOI:** 10.3389/fmicb.2017.01805

**Published:** 2017-09-20

**Authors:** Litty Babu, Siva R. Uppalapati, Murali H. Sripathy, Prakash N. Reddy

**Affiliations:** ^1^Department of Microbiology, Defence Food Research Laboratory Mysore, India; ^2^Department of Biotechnology, Vignan’s Foundation for Science, Technology and Research University Guntur, India

**Keywords:** *Klebsiella pneumoniae*, subunit vaccine, OmpA, OmpK36, animal challenge

## Abstract

Safety and protective efficacy of recombinant multi-epitope subunit vaccine (r-AK36) was evaluated in a mouse model. Recombinant AK36 protein comprised of immunodominant antigens from outer membrane proteins (Omp’s) of *Klebsiella pneumoniae* namely OmpA and OmpK36. r-AK36 was highly immunogenic and the hyperimmune sera reacted strongly with native OmpA and OmpK36 proteins from different *K. pneumoniae* strains. Hyperimmune sera showed cross-reactivity with Omp’s of other Gram-negative organisms. Humoral responses showed a Th2-type polarized immune response with IgG1 being the predominant antibody isotype. Anti-r-AK36 antibodies showed antimicrobial effect during *in vitro* testing with MIC values in the range of 25–50 μg/ml on different *K. pneumoniae* strains. The recombinant antigen elicited three fold higher proliferation of splenocytes from immunized mice compared to those with sham-immunized mice. Anti-r-AK36 antibodies also exhibited *in vitro* biofilm inhibition property. Subunit vaccine r-AK36 immunization promoted induction of protective cytokines IL-2 and IFN-γ in immunized mice. When r-AK36-immunized mice were challenged with 3 × LD_100_ dose, ∼80% of mice survived beyond the observation period. Passive antibody administration to naive mice protected them (67%) against the lethal challenge. Since the targeted OMPs are conserved among all *K. pneumoniae* serovars and due to the strong nature of immune responses, r-AK36 subunit vaccine could be a cost effective candidate against klebsiellosis.

## Introduction

*Klebsiella pneumoniae* is a Gram-negative opportunistic pathogen that belongs to family Enterobacteriaceae. It is the most common cause of nosocomial respiratory tract infections and second most frequent cause of bacteraemia and urinary tract infections (Center for Disease Control [CDC], 1974; Ahmad et al., 2012; Huang et al., 2015). *K. pneumoniae* is also an important pathogen causing severe morbidity and mortality (>50%) in intensive care units, pediatric and surgical wards (Rice et al., 1990; Coovadia et al., 1992; Palusiak, 2015). *K. pneumoniae* is recognized as a major health threat worldwide and the treatment options of *Klebsiella* infections are limited owing to high incidence of multiple drug resistance and adverse antibiotic reactions (Breecher, 2007; Vieira et al., 2016). A novel and distinct *K. pneumoniae* variant called hypervirulent *K. pneumoniae* (hvKP) has emerged which is predicted to become a major threat in Asia and Western countries (Diago-Navarro et al., 2017). Infections caused by such strains are reported to be associated with higher morbidity and mortality even with antibiotic sensitive strains. In comparison to classical *K. pneumoniae*, hvKP strains exhibit enhanced virulence in terms of over production of CPS, possess anti-phagocytosis and severe metastases, cause severe infections such as pyogenic liver abscess, pneumonia and endophthalmitis (Wang et al., 2017). Management of hvKP infections is going to be extremely challenging due to multidrug resistance including acquisition of extended-spectrum β-lactamases, carbapenemases and recently described NDM-1 (Elemam et al., 2009). Control of *K. pneumoniae* is also hampered by additional mechanisms such as endotoxin production that induces septic shock, CPS that inhibits phagocytosis and resistance to compliment-mediated killing (Merino et al., 1992; Podschun and Ullmann, 1998; Kim et al., 2002). This has directed attention toward controlling *K. pneumoniae* infections through bacterial clearance from body employing alternate strategies to improve immune defenses such as probiotics (Vieira et al., 2016) or more specific means such as vaccination and antibody therapy (Jain et al., 2015; Lee et al., 2015).

At present, the knowledge regarding the pathogenic mechanisms utilized by *K. pneumoniae* to develop an infection is limited. However, five main classes of virulence factors were identified namely capsule, LPS, siderophores, adhesins (pili, fimbriae, OMPs) and exotoxins. *K. pneumoniae* might not be simple to control by regular vaccine approaches due to its high degree of antigenic variation among strains including CPS and LPS antigens. Clearance of *K. pneumoniae* from the host system requires effective host defense mechanisms, to which bacterial surface plays a major role. Three components of bacterial surface are suspected in the development of immunity: CPS, LPS and OMPs. Capsular antigens of *K. pneumoniae* have been implicated as important virulence factors and help the bacteria in preventing the killing by serum and escaping from the host immune system by avoiding phagocytosis. Studies have indicated a direct correlation between capsule size and pathogenicity (Simoons-Smit et al., 1986). Till date common vaccines used against *Klebsiella* infections are based on CPS and also LPS to a certain extent (Cryz, 1983, 1990; Yadav et al., 2005). However, CPS and LPS provide only type-specific protection against these infections since at least seventy seven capsular (K) antigen types and eight LPS (O) antigen serotypes exist. OMPs are surface proteins which exist as trimers and act as water-filled channels that allow the hydrophilic molecules across the membrane. These are involved in antibiotic resistance mechanisms and contribute to the virulence of the organism. OMPs of *K. pneumoniae* are considered safe as subunit vaccines and have been tested and shown to induce host specific antibodies and found effective without the need of additional adjuvants in animal models (Sun et al., 2014). OmpK36 is produced by the majority of extended-spectrum beta-lactamase-producing (ESBL) *K. pneumoniae* members and it is also reported to contribute to resistance or reduced susceptibility to carbapenems in ESBL-producing *K. pneumoniae* strains (Mena et al., 2006; Skurnik et al., 2010). OmpA is one of the major proteins of outer membranes of Gram-negative bacteria and is highly conserved among the members of Enterobacteriaceae (Pichavant et al., 2003). OmpA has been shown to possess adjuvant properties and also induce specific humoral and anti-tumor cytotoxic responses when coupled with tumor antigens (Jeannin et al., 2002). Individually OmpA and OmpK36 as DNA vaccines were shown to confer protective immune responses in mouse model (Kurupati et al., 2011). Therefore, OmpA and OmpK36 could be ideal targets for design of anti-*Klebsiella* vaccine since these targets are carried by all the strains unlike O and K antigens.

For effective control of *K. pneumoniae* infections in the wake of widespread multiple antibiotic resistance mechanisms, preventive measures by active and passive immunization approaches appear to be promising. Therefore, in the present study we constructed a chimeric gene comprising immunodominant regions of OmpA and OmpK36 fused together by a glycine linker followed by evaluation of its protective efficacy in BALB/c mouse model. Hyperimmune serum from immunized mice was characterized for its *in vitro* neutralization ability against virulent *K. pneumoniae* strains and *in vivo* protective efficacy against lethal challenge.

## Materials and Methods

### Materials

Dehydrated media and supplements were procured from HiMedia laboratories, India. *Taq* DNA polymerase, dNTPs, IPTG and adjuvants were from Sigma–Aldrich, India. *Pfu* DNA polymerase was from Fermentas, United States. pRSET vector series, *Escherichia coli* DH5α and BL21DE3 strains are from Invitrogen, United States. *K. pneumoniae* ATCC 13883 and ATCC 10031 strains were procured from MicroBiologics Inc., United States. All the cell culture reagents were from Sigma–Aldrich, India. Specific pathogen-free BALB/c mice used in the study were received from Central Animal Housing Facility of Defence Food Research Laboratory (DFRL), Mysore city, India.

### Bacterial Strains

Genomic DNA of *K. pneumoniae* ATCC 13883 strain was used for amplification of *ompK36* and *ompA* genes for chimeric gene construction. Additionally, *K. pneumoniae* E-1716 a clinical isolate belonging to K1 serotype isolated from sputum sample collected from a patient admitted at Sri Dharmasthala Manjunatheshwara (SDM) College of medical science and hospital, Dharwad, India was used for challenge experiments based on its higher virulence (lower LD_50_ values) potential. For animal experiments, *K. pneumoniae* was cultured from glycerol stocks in BHI broth and incubated overnight at 37°C with vigorous shaking. Following centrifugation, bacterial cells were washed two times and suspended in 1x PBS solution. LD_50_values were determined by intraperitoneal administration of different serial dilutions of three *K. pneumoniae* strains.

### Cloning, Expression and Purification

Primers were designed for amplification of *ompA* and *ompK36* genes based on gene sequences available at GenBank (**Table 1**). The individual genes were fused using PCR by ‘gene splicing by overlap extension’ (SOE-PCR) method employing the primers shown in **Table 1**. Restriction sites (*Bam*HI and *Hind* III) were added to 5′ end of primers so as to clone the resulting fusion gene in-frame with the coding sequence of pRSETA vector. The recombinant plasmid was transferred to *E. coli* DH5α and BL21(DE3) strains. A single transformed BL21(DE3) clone was subjected to induction with IPTG followed by testing for chimeric protein expression by SDS-PAGE analysis and Western blotting with anti-histidine antibodies. For bulk purification, recombinant clone was cultured in 500 ml Luria-Bertani broth followed by purification of histidine-tagged (6xHis) protein using Ni-NTA resin (Qiagen) by gravity flow protocol as per manufacturer’s instructions.

**Table 1:** List of primers, their sequences and accession numbers used for construction of chimeric rAK36 gene.

Primer	Sequence (5′–3′)	Accession number	Product size	Fusion gene
			(bp)	(bp)
ompK-F-*BamH*I	CGC***GGATCC***GGCGGCTGAAATTTATAACAA	NC_016845.1	1044	1587
ompK-R-Linker	***^∗^AGAACCACCACCACCGGAGCCGCCG***			
	**CCGCC**CACGTCGTCGGTAGAGATAC			
ompA-F-Linker	***^∗^GGTGGTGGTGGTTCTGGCGGCGGCG***	AJ000998.1	558	
	***GCTCC***ACCTGGTATGCAGGTGGTA			
ompA-R-*Hind*III	GGG***AAGCTT***ACCGAAGCGGTAGGAAAC			

*Bold and italicized letters represents the restriction enzyme recognition sites for directional cloning.*

*^∗^Bold, italicized, and underlined letters represents the DNA sequences corresponding to complementary glycine linker sequences for splicing of two genes by SOE-PCR.*

### Immunization and Hyperimmune Sera Generation

Six to seven-week-old female BALB/c mice were grouped into three batches of 10 mice each. Blood was drawn from all the mice 2 days prior to the start of immunization schedule and the pooled serum served as negative control in all the experiments. Two groups of mice received 50 μg of recombinant protein subcutaneously in emulsion with Freund’s complete adjuvant followed by three booster doses at 10 days interval with the same quantity of protein in Freund’s incomplete adjuvant. Third group served as a control receiving only adjuvants in 1x PBS by intraperitoneal route. Blood was drawn 2 days prior to first immunization, 1 day prior to the second booster and 7 days past the last immunization and the pooled sera were used for evaluation of antibody titers and isotypes. Of the 20 immunized mice, two sets of 6 mice each were used in active challenge and memory groups. Three mice were used for splenocyte stimulation experiments. Remaining mice served as a backup in case of accidental deaths. Of the 10 sham-immunized mice, 6 were used in active challenge and 3 were used for splenocyte proliferation assay. For passive immunization and challenge naïve mice were used.

### Humoral Response and Antibody Isotyping

Antibody titers of the pooled serum were measured after the second and the third booster immunizations by indirect ELISA employing r-AK36 protein as antigen. Maximum dilution at which the mean absorbance (OD_600_) was at least two times more or equals the mean value of negative control was considered as endpoint titers. Serum samples were diluted 1:500 in PBS followed by antibody isotype determination employing the mouse monoclonal antibody isotyping reagents (Sigma, United States) as per manufacturer’s recommendations. Sham-immunized serum served as controls during isotype determination.

### Western Blot Analysis

Western blot analysis was performed to test the reactivity of r-AK36 hyperimmune sera with native OmpA and OmpK36 proteins from various *K. pneumoniae* strains along with other Enterobacteriaceae members. Whole-cell lysates of various bacterial strains were separated on 12% SDS-PAGE gels followed by electrotransfer on to nitrocellulose membrane. Membranes were blocked in 5% skim milk solution followed by washing in PBS-Tween 20 (0.05%) and probing with 1:1000 dilutions of anti-r-AK36 sera. After washing, membranes were treated with 1:3000 dilutions of goat anti-mouse IgG conjugated with horseradish peroxidase (HRP). Blots were developed either with diaminobenzidine tetrahydrochloride (Sigma–Aldrich, India) and 30% H_2_O_2_ or ready to use tetramethylbenzidine (Bangalore Genei, India) solution.

### Lymphocyte Proliferation Assay and Cytokine Analysis

Seven days after the last immunization, aseptically isolated spleens from immunized and sham-immunized mice were gently ruptured on a wire mesh using sterile glass rod with round edges to break splenic capsule and separating connective tissue followed by flushing with sterile DMEM. The resultant splenocytes were treated with ACK buffer (150 mM NH_4_Cl, 10 mM KHCO_3_ and 0.1 mM EDTA) to remove RBCs. The splenocytes were resuspended in Dulbecco’s Modified Eagle’s medium (DMEM) supplemented with 10% heat-inactivated fetal bovine serum (Hyclone, Thermo scientific, United States), 5 mM glutamine, 50 U/ml penicillin, 50 μg/ml of streptomycin and 0.2% NaHCO_3_. This experiment was performed three times with three mice from active and sham-immunized groups. The number of splenocytes used for the stimulation studies was 10^6^ cells/well. The antigen (recombinant protein) was used at a concentration of 2, 4, 6, 8, and 10 μg/well. Concanavalin A (Sigma–Aldrich, United States) was included as a mitogen for positive control at a concentration of 10 μg/ml. The experiment was performed in triplicates. The plates were incubated at 37°C in 5% CO_2_ for 72 h. The proliferation was measured using MTT reagent (Sigma, India) as described elsewhere (Reddy et al., 2015). Lymphocyte proliferation was calculated as stimulation index employing the following formula: Stimulation index = OD_570_ with antigen/OD_570_ without antigen. The supernatants from cultured splenocytes were evaluated for cytokine estimation (IL-2, IL-4, IL-10, IFN-γ, and TNF-α) employing commercial kits (MabTech, Nacka, Sweden) based on sandwich ELISA.

### *In Vitro* Antimicrobial Susceptibility Testing

To determine the antibacterial activity of r-AK36 antibodies against *K. pneumoniae*, microtiter plate-based *in vitro* antimicrobial susceptibility test was carried out as per CLSI recommendations. In brief, 1 mg/ml of antibody stock was serially diluted in PBS to 100, 75, 50, 25, and 10 μg/ml concentrations and 100 μl volumes of each dilution were tested for antimicrobial activity in triplicates. Approximately 100 μl of 10^6^/ml CFU of *K. pneumoniae* ATCC 13883, *K. pneumoniae* ATCC 10031 and *K. pneumoniae* E-1716 strain diluted in double-strength Mueller-Hinton broth were added to each antibody dilution in three replicates. Only the PBS with no antibody and *K. pneumoniae* in MH broth served as negative control. Only PBS along with MH broth served as a blank. Rifampicin antibiotic served as positive control during antimicrobial testing. Absorbance at OD_600_ was recorded before the start of experiment followed by incubation of plates at 37°C overnight. The lowest antibody dilution at which there was no increase in turbidity after overnight incubation was considered as MIC of the polyclonal antibodies.

### Biofilm Inhibition Assay

Effect of sub-inhibitory concentrations of r-AK36 antibodies on biofilm formation was assessed by microtiter plate method as described by Sun et al. (2005) with minor modifications. *K. pneumoniae* standard and clinical isolates were grown overnight in BHI broth at 37°C. Bacterial cells were harvested and the cell count was adjusted to 10^6^ CFU/ml using sterile double-strength BHI broth (with 1% glucose) and 100 μl was mixed with 100 μl of 50 μg/ml polyclonal antibodies and incubated at 37°C overnight with shaking. Post-incubation, plate was washed two times in PBS to remove non-adherent cells. Adherent biofilm was fixed by adding 200 μl of methanol for 10 min. After drying the plates, 250 μl of 1% crystal violet was added to each well and incubated for 5 min. Plates were washed under running water, dried and the adherent biofilm was solubilised by adding 250 μl of 33% glacial acetic acid. OD was measured at 570 nm in Tecan multimode plate reader. A control was included for each strain with only BHI broth and glucose without antibodies. Each sample was tested in triplicates. The percent inhibition of biofilm formation of each *K. pneumoniae* strain was determined using the formula: (OD_570_ in control – OD_570_ in treatment)/(OD_570_ in control) × 100.

### Challenge Studies

For studying the protective efficacy of the recombinant protein, the mice were challenged with a hypervirulent strain (K1 serotype) of *K. pneumoniae* whose lethal dose (LD_50_) had been determined to be 3 × 10^5^ CFU. Two groups of six mice each of both immunized and sham-immunized were challenged intraperitoneally 10 days after the last immunization with 10^6^ CFU in 100 μL of 0.9% saline. The protective efficacy of the recombinant protein was checked by evaluating the survival rates after 15 days of challenge. Every day the animals were observed for signs of illness, such as weight loss, change in coat appearance, and lack of movement. On day 15 the remaining mice were terminated to collect blood for antibacterial assay and organs were collected for testing bacterial load. Similar challenge experiments were performed to test the persistence of immunization and memory response 120 days after the last immunization and observed for survival rates.

### Bacterial Load in Organ Systems

After the 15 days observation period following bacterial challenge, two mice were humanely euthanized by CO_2_ sedation followed by cervical dislocation. Before excising the organs, the surface of the viscera was swiped with sterile cotton swabs to check the bacterial translocation and cultured in BHI broth. Further kidney, spleen, liver, and lungs were excised and transferred to sterile preweighed Eppendorf tubes. The tissues from organs were macerated by plastic tissue grinder and the resultant homogenate and blood samples were plated on plate count agar. The colony count was measured following incubation at 37°C for 24 h.

### Passive Immunotherapy

The *in vivo* prophylactic and neutralization efficacy of the hyperimmunepolysera was determined by performing passive antibody administration where in 6-week-old naïve female BALB/c mice (*n* = 6) were injected intraperitoneally with 200 μL of polysera and left for 24 h followed by challenge with 2 × LD_100_ (4 × 10^5^ CFU) of *K. pneumoniae* E-1716 strain. A control group (*n* = 6) was injected with same quantity of PBS alone followed by challenge with same quantity of lethal dosage. Mice were observed for a period of 14 days for any possible deaths. Survival of mice post-challenge during the observation period is represented by Kaplan–Meier graphs using GraphPad Prism 5.0 version.

### Statistical Analysis

The data were presented either as mean ± SD or individual values+grand median. Mantel-Cox (log-rank) test was used to compare the survival curves and Student’s *t*-test was used for other statistical comparisons. All graphical illustrations were constructed by GraphPad Prism 5 software. Statistical difference between different groups were analyzed by univariate ANOVA using Minitab 17. Significance (*P*)-value summary: ^∗^*P* ≤ 0.05, ^∗∗^*P* ≤ 0.01, ^∗∗∗^*P* ≤ 0.001; no significance #.

### Animal Ethics

All the animal experiments, biosafety procedures and genetic manipulations undertaken in this study have been performed with permission and following the guidelines laid by the Institutional Animal Ethics Committee (IAEC) and the Institutional Bio-Safety Committee (IBSC) of Defence Food Research Laboratory, Mysore. Permission for using the experimental animals was approved by Institutional Animal Ethical Committee during the meeting held on 4th December, 2015 at DFRL, Mysore. Animals were received from DFRL animal housing facility (28/1999/CPCSEA dt. 11 March, 1999) and maintained under hygienic conditions in animal house of Microbiology Department of DFRL, Mysore. The clinical isolate used for challenge experiments in this study was received as pure culture from the culture collection of SDM hospital after taking mutual consents of Head of microbiology departments of SDM hospital and DFRL. Direct sampling of clinical specimens was not carried out in this study. The IAEC of DFRL approved the protocols employed.

## Results

### Synthesis of Chimeric Recombinant Protein r-AK36

**FIGURE 1:**
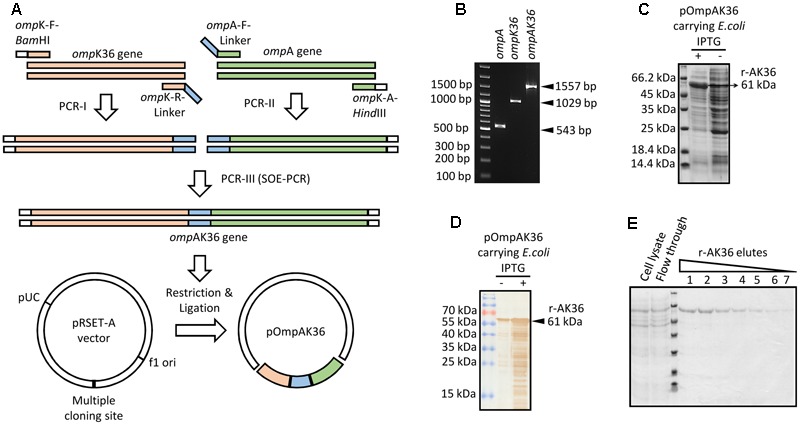
Cloning and expression of r-AK36 fusion protein in pRSETA vector. **(A)** Schematic representation of PCR amplification, splicing and cloning of chimeric r-AK36 gene in pRSETA vector. **(B)** Agarose gel showing PCR amplified *ompA* and *ompK36* genes and *ompAK36* spliced gene. **(C)** Stained SDS-PAGE gel showing expressed rAK36 protein upon IPTG induction. **(D)** Western blot probed with anti-histidine antibodies showing 6x-His tagged r-AK36 protein. **(E)** SDS-PAGE gel showing r-AK36 protein from different elutions following IMAC purification with Ni-NTA agarose.

Fusion gene comprising immunodominant regions of *K. pneumoniae ompA* and *ompK36* genes was generated by SOE-PCR (**Figures 1A,B**) and cloned into pRSETA vector. The chimeric r-AK36 gene was sequenced and was found not to possess any deletions or point mutations when compared to the expected sequence. Transformation of recombinant plasmid in *E. coli* host was followed by induction with IPTG which resulted in the expression of a protein at 61 kDa region as observed in SDS-PAGE (**Figure 1C**) and Western blot analysis (**Figure 1D**). This was in accordance with the predicted size of the protein deduced from the fusion gene sequence in vector. Histidine-tagged (6xhis) recombinant protein in inclusion bodies was extracted and purified from 500 ml induced culture by immobilized metal affinity chromatography under denaturing conditions (**Figure 1E**). Purified protein was subjected to step dialysis with decreasing concentrations of urea under reducing environment. Final protein concentration was quantified and adjusted to 2.5 mg/ml by Lowry’s calorimetric assay against BSA standard.

### Immunogenicity of r-AK36 in BALB/c Mice

**FIGURE 2:**
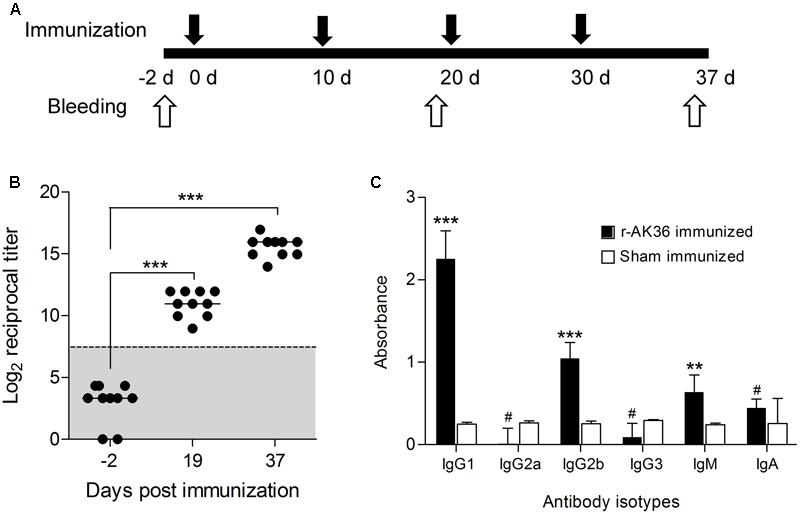
Determination of antibody titers and antibody isotypes from r-AK36 evoked anti-r-AK36 serum in BALB/c mice. **(A)** Schematic representation of immunization and blood collection schedules of r-AK36 antigen. **(B)** Estimation of antibody titers of anti-r-AK36 serum induced by r-AK36 subunit vaccine at different time points of immunization. **(C)** Antibody isotypes induced by r-AK36 was evaluated by ELISA using isotype-specific monoclonal antibodies for IgG1, IgG2a, IgG2b, IgG3, IgM and IgA. Data are represented in mean ± SD (*n* = 8).Significance (*P*)-value summary analyzed by univariate ANOVA: ^∗^*P* ≤ 0.05, ^∗∗^*P* ≤ 0.01; ^∗∗∗^*P* ≤ 0.001; no significance #.

In immunized mice, a progressive and significant increase of anti-r-AK36 antibodies was observed. After three booster doses (**Figure 2A**), the end-point antibody titers reached 1:64000, while the control sera from sham-immunized mice showed no reactivity with r-AK36 in indirect ELISA (**Figure 2B**). Pooled sera from r-AK36-immunized mice, when subjected to isotype analysis, a Th2-type immune response was observed indicated by the predominance of IgG1 immunoglobulin. Surprisingly, IgG2b levels also predominated the immune response indicating Th1 polarization. IgM antibodies also persisted in serum even after last immunization and Ig class switching (**Figure 2C**). Despite significant IgG2b levels, the relative degree of contribution by Th2- and Th1-type humoral immune responses determined as IgG1:IgG2a ratio is >1, indicating r-AK36 in CFA/IFA adjuvant system induced primarily Th2-type antibody response in BALB/c mice.

**FIGURE 3:**
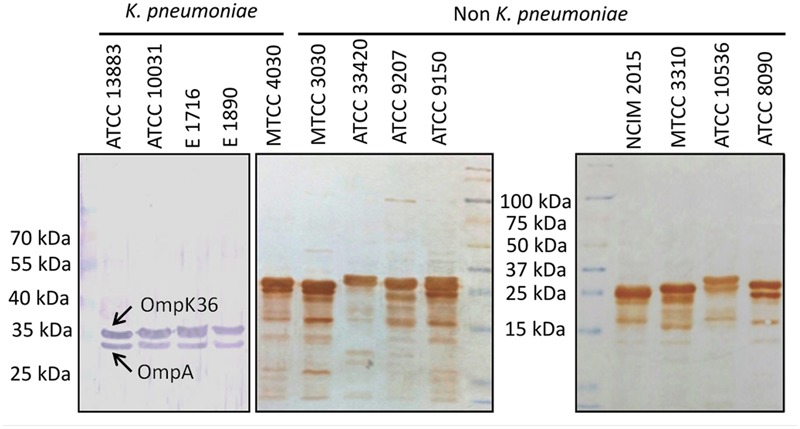
Western blot analysis for testing reactivity of anti-r-AK36 antibodies with different *Klebsiella pneumoniae* and related Enterobacteriaceae members. Ant-r-AK36 serum reacted clearly with native OmpA and OmpK36 proteins of *K. pneumoniae* strains, while same serum cross-reacted with other members of Enterobacteriaceae at different regions.

The specific reactivity of anti-r-AK36 antibodies with the native OMPs of various *K. pneumoniae* and other bacterial species was assessed by Western blot analysis. Anti-r-AK36 antibodies showed strong reactivity with native OmpA (37 kDa) and OmpK36 (39.6 kDa) proteins from different *K. pneumoniae* standard and isolated strains (**Figure 3**). Other Gram-negative rods of Enterobacteriaceae family showed varying reactivities at different molecular sizes. This is not surprising considering the conserved nature of epitopes on OmpA and OmpK36 proteins among Enterobacteriaceae members. Reactivities of anti-r-AK36 antibodies with different bacterial species are provided in **Table 2**.

**Table 2:** List of bacterial strains tested for reactivity with mouse anti-rAK36 sera.

Sl. no.	Bacterial strain	Reaction	
		OmpA	OmpK36
(1)	*K. pneumoniae* ATCC-13883	+	+
(2)	*K. pneumoniae* ATCC-10031	+	+
(3)	*K. pneumoniae* E-1716	+	+
(4)	*K. oxytoca* MTCC-3030	+	+
(5)	*Escherichia coli* ATCC-10536	+	+
(6)	*E. coli* MTCC- 1687	+	+
(7)	*Salmonella paratyphi A* ATCC-9150	+	+
(8)	*S. typhimurium* ATCC-13311	+	+
(9)	*Shigella flexneri* ATCC-9199	+	+
(10)	*Sh. boydii* ATCC-9207	+	+
(11)	*Enterobacter aerogenes* MTCC-2693	+	+
(12)	*Citrobacter freundii* ATCC-8090	+	+
(13)	*Proteus vulgaris* ATCC-33420	+	+
(14)	*P. mirabilis*MTCC-3310	+	+

### *In Vitro* Proliferation of Lymphocytes from r-AK36-Immunized Mice and Cytokine Analysis

**FIGURE 4:**
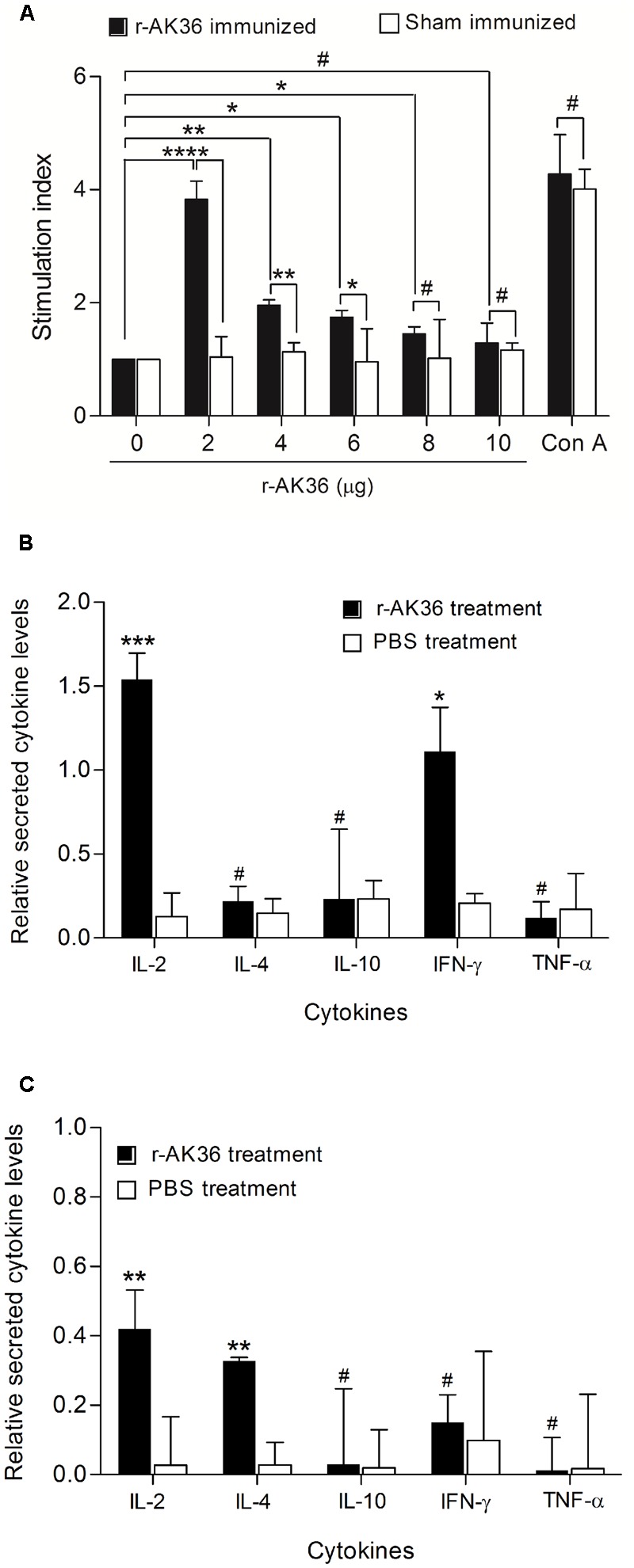
Lymphocyte proliferation and cytokine analysis from splenocytes of r-AK36-immunized and sham-immunized mice. **(A)** Proliferation of lymphocytes isolated from r-AK36-immunized and sham-immunized BALB/c mice and treated with r-AK36 protein at different concentrations for 72 h. **(B)** Bar graphs showing mean cytokine levels relative to positive controls in conditioned media of splenocytes isolated from r-AK36 immunized BALB/c mice, after stimulation for 3 days with r-AK36 protein. **(C)** Bar graphs showing mean cytokine levels relative to positive controls in conditioned media of splenocytes isolated from sham-immunized BALB/c mice, after stimulation for 3 days with r-AK36 protein. All results are the average of three independent experiments. Significance (*P*)-value summary analyzed by univariate ANOVA: ^∗^*P* ≤ 0.05, ^∗∗^*P* ≤ 0.01, ^∗∗∗^*P* ≤ 0.001, ^∗∗∗∗^*P* ≤ 0.0001; no significance #.

The r-AK36 antigen induced a significant proliferation of splenocytes (whole lymphocytes) from r-AK36-immunized mice upon reinduction by the same antigen at a concentration of 4 μg per million cells. When splenocytes from sham-immunized mice were induced with r-AK36, no significant proliferation was observed (**Figure 4A**). As a positive control, 10 μg/well of Concanavalin A was used and a significant increase in proliferation was observed in the lymphocyte population, confirming the efficiency of the cell purification process.

The Th2polarization of immune response suggested a role of IL-4 lymphokine in mediating IgG1 production. Also, significant proliferative index observed in r-AK36-immunized mice splenocytes prompted us to assess the levels of pro-inflammatory and anti-inflammatory cytokines from conditioned media after lymphocyte proliferation. No increase in the production of IL-4 cytokine was observed from r-AK36-immunized mice splenocytes but a significant rise in the same was observed when sham-immunized mice splenocytes were induced with r-AK36 protein (**Figures 4B,C**). On the other hand, IL-2, a cytokine that promotes differentiation of T-cells to effector and memory T-cells was significantly produced from r-AK36-immunized mice. IL-2 plays a role in the development of T cell immunologic memory by expanding the number and function of antigen-selected T cell clones. Also, IFN-γ was found to be produced significantly from the r-AK36-immunized mice splenocytes (**Figure 4B**). IFN-γ was found to be crucial for controlling pulmonary pneumoniae caused by *K. pneumoniae*. IFN-γ is involved in host immunological defense against *K. pneumoniae* infection (Yoshida et al., 2001). Splenocytes from sham-immunized mice upon induction by r-AK36 produced significant levels of IL-2 cytokine (**Figure 4C**).

### Challenge Studies

To challenge the immunized mice with *K. pneumoniae*, firstly we ventured into determining LDs of various strains. The LD (LD_100_ and LD_50_) of different *K. pneumoniae* strains varied significantly (**Table 3**). In general, these values were lower for strains recovered from clinical sources rather than the standard cultures indicating their higher virulence potential. Groups of 4 mice were used for each dilution during LD determination. *K. pneumoniae* E-1716 strain isolated from sputum sample from an infected patient had lower LD_50_ values than *K. pneumoniae* ATCC 13883 and ATCC 10031 strains. The clinical strain was also different in terms of pathology induced post-challenge and the rapidity of mortality. The clinical strain induced acute respiratory distress, lethargy and rapid death between 24 and 48 h of pathogen administration. On the other hand, mice challenged with standard strains showed reduced activity and dullness in movement and delayed death beyond 48 h of administration.

**Table 3:** Lethal dose determination for various *K. pneumoniae* strains in BALB/c mouse model.

Sl. No.	Bacterial strain	LD_100_	LD_50_
(1)	*K. pneumoniae* E- 1716	3 × 10^5^	1.5 × 10^5^
(2)	*K. pneumoniae* ATCC- 10031	4 × 10^6^	2 × 10^6^
(3)	*K. pneumoniae* ATCC- 13883	6 × 10^6^	3 × 10^6^

**FIGURE 5:**
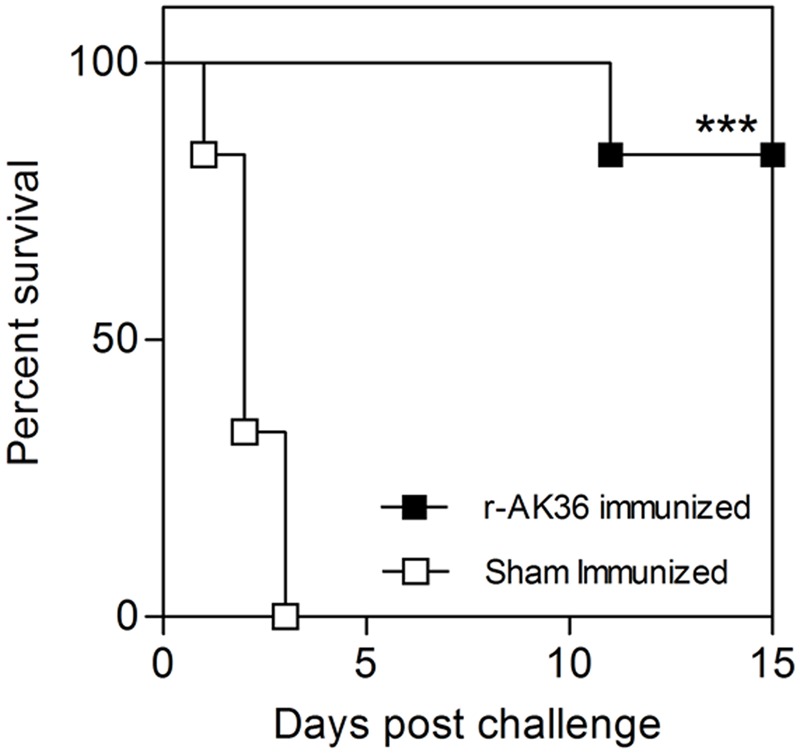
Challenge studies in r-AK36-immunized mice. Kaplan–Meier’s survival graph for r-AK36-immunized and sham-immunized mouse challenged with LD of *K. pneumoniae* E-1716 strain. One animal succumbed to death (83% survival) in active immunized group, but none survived in sham-immunized group. Significance (*P*)-value summary: ^∗∗∗^*P* ≤ 0.001.

Groups of six r-AK36-immunized and sham-immunized mice were challenged with ∼10^6^ CFU (3 × LD_100_) of virulent *K. pneumoniae* E-1716 strain by intraperitoneal route 7 days after the last booster dose immunization. At least 83% of mice survived beyond the observation period with only one death on day 11 (**Figure 5**). None of the sham-immunized mice survived beyond 72 h indicating the protective role of r-AK36 subunit vaccine. Gross pathology of lungs of challenged sham-immunized mice showed enlarged, swollen and dull gray regions affecting entire lobes with edema.

### Antibacterial Activity of Anti-r-AK36 Antibodies

**FIGURE 6:**
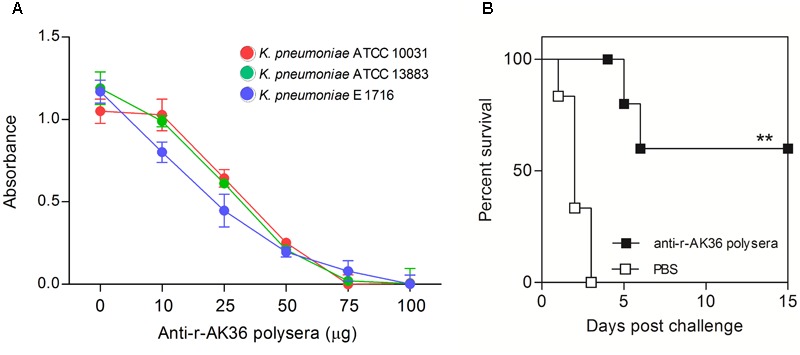
Determination of MIC values of anti-r-AK36 serum and challenge studies on passive immunized mice. **(A)** MIC values were found to be in the range of 25–50 μg/ml of polyclonal antibodies against different strains. **(B)** Survival graph showing passively immunized mice challenged with r-AK36 antigen. Two mice succumbed to death (67% survival) from passive immunized mice compared all six from sham-immunized group. Significance (*P*)-value summary: ^∗∗^*P* ≤ 0.01.

To assess the antibacterial activity of anti-r-AK36 antibodies, three assays were utilized, viz., MIC, biofilm inhibition and antibacterial protection conferred by passive transfer of antibodies. The MIC values were moderately uniform over a different range of *K. pneumoniae* strains. The MIC value of r-AK36 antibodies collected 7 days past the last booster dose immunization was determined to be in the range of 60–100 μg/ml. The inhibition of bacteria is also time and concentration dependant as seen from the **Figure 6A**.

Antibodies against surface proteins can inhibit biofilm formation by coating the pathogens surface and targeting for destruction. Anti-r-AK36 antibodies at subinhibitory concentrations reduced the ability of different *K. pneumoniae* strains belonging to various serotypes to form biofilm *in vitro*. Since the ability of *K. pneumoniae* to form biofilm varies between strains, a preliminary test was carried out to ascertain the biofilm forming potential. The percent reduction of biofilm varied significantly between different strains. The amount of biofilm formation quantified by crystal violet staining with and without addition of antibody and the percent inhibition of biofilm formation is provided in **Table 4**.

**Table 4:** Biofilm inhibition assay on *K. pneumoniae* by OmpAK36 antibodies.

Sl.	Strain name	Without	With	Percent
No.		antibodies	antibodies	inhibition
(1)	*K. pneumoniae* ATCC-10031	1.547	0.392	74.6
(2)	*K. pneumoniae* E-1716	1.714	0.48	71.9
(3)	*K. pneumoniae* E-1764	1.146	0.289	74.7
(4)	*K. pneumoniae* E-1772	1.284	0.491	61.7
(5)	*K. pneumoniae* E-2013	1.372	0.295	78.4
(6)	*K. pneumoniae* E-1997	1.568	0.573	57.3
(7)	*K. pneumoniae* E-1766	1.772	0.586	66.9
(8)	*K. pneumoniae* E-1773	1.095	0.243	77.8
(9)	*K. pneumoniae* E-1845	1.543	0.241	84.3
(10)	*K. pneumoniae* E-1785	1.244	0.381	69.3
(11)	*K. pneumoniae* E-1890	1.289	0.297	76.9
(12)	*K. pneumoniae* E-2076	1.351	0.394	70.8

Passive administration of anti-r-AK36 antibodies protected the mice significantly from mortality upon challenge by 4 × 10^5^ CFU of *K. pneumoniae* E-1716. Approximately 67% of mice survived beyond the observation period post-challenge (**Figure 6B**).

### Immunological Memory Assessment

**FIGURE 7:**
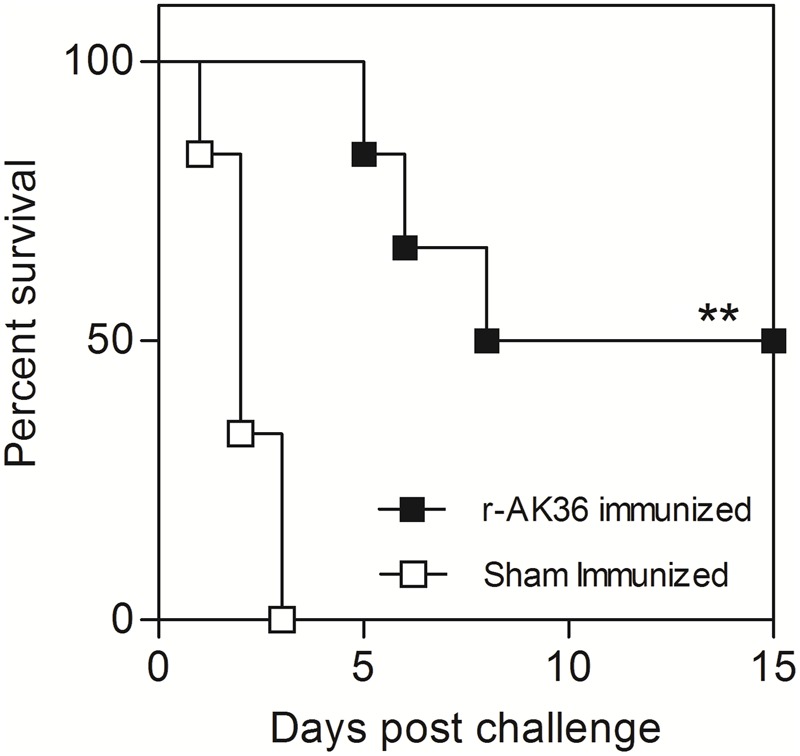
Challenge studies in r-AK36-immunized mice 120 days post last immunization. Three mice succumbed to death (50% survival) in active immunized mice while no survivors were observed in sham-immunized group of mice. Significance (*P*)-value summary: ^∗∗^*P* ≤ 0.01.

Persistence of immunity was evaluated by subjecting r-AK36-immunized mice to lethal challenge with *K. pneumoniae* E-1716 strain 120 days past final booster. Approximately 50% of the challenged mice survived beyond the observation period in memory group (**Figure 7**).

Viable bacterial counts from various organs such as lungs, spleen, kidney, and liver were evaluated from challenged mice sacrificed after the observation period. Results indicated that lungs of sham-immunized mice showed higher infiltration of *K. pneumoniae* while lungs of r-AK36-immunized mice showed significantly reduced bacterial counts. The same trend in CFU counts was seen with other organs namely kidney, liver, and spleen. Bacterial infiltration was highest in lungs followed by liver, kidney, and colon (**Table 5**).

**Table 5:** Viable bacterial counts for tissues from different experimental groups of challenged mice.

Organs	Immunized		Unimmunized (following death) (CFU/organ)
	Post 15 days (CFU/organ)	Post 100 daysfrom survivors	
Lungs	0.58 × 10^2^ ± 0.12 × 10^2^	0	4.2 × 10^5^ ± 6.5 × 10^2^
Kidney	0.23 × 10^2^ ± 0.09 × 10^2^	0	2.8 × 10^5^ ± 4.8 × 10^2^
Liver	0.32 × 10^2^ ± 0.12 × 10^2^	0	3.1 × 10^5^ ± 1.2 × 10^3^
Intestine	0.12 × 10^2^ ± 0.04 × 10^2^	0	1.2 × 10^5^ ± 3.6 × 10^2^

*Values represent mean ± SD of two independent readings (*n* = 2).*

## Discussion

Multidrug-resistant *K. pneumoniae* is responsible for a variety of nosocomial and community acquired infections. Clinical trials addressing MDR infections are focused mainly on antimicrobials, neglecting vaccine development. Efforts onto discovery of non-traditional approaches to combat *K. pneumoniae* have been hampered by poor knowledge of immunological correlates. On-going investigations on preclinical studies have shown that Th17-mediated immunity can confer broad protection against MDR Gram-negative infections. Approaches to enhance host immunity and for reducing bacterial infection such as adenoviral gene therapy for over production of IL-2, using TNF-α pro-inflammatory cytokine and cyclic di-GMP have shown promise in boosting immunity and reduced bacterial infections. But none of these molecules could reach market due to their high price, being non-specific and no clinical study support. In these difficult times, promising alternatives to antibiotics seems to be pathogen-specific active vaccination and passive antibody therapy (Babb and Pirofski, 2017). Th17 cells are shown to confer serotype independent immunity by recognizing conserved antigens among Enterobacteriaceae members. A recombinant OmpX protein cloned from *K. pneumoniae* was recognized by *Klebsiella* immune serum and Th17 cells from *Klebsiella* immunized mice. Intranasal administration of purified r-OmpX induced strong mucosal Th17 responses necessary for protection against infectious diseases (Chen et al., 2014). Intranasal administration of mouse with heat-killed *K. pneumoniae* induced IL-17 production in lungs and conferred protection in immunized mice during live *K. pneumoniae* challenge. It was also reported that oral immunization reduced the bacterial burden during pulmonary infection through recruitment of Th17 cells (Chen et al., 2011a). Among the recently reported immunotherapies, one group reported that monoclonal antibodies generated against the antigen MrkA, a major protein in fimbrial III complex reduced biofilm formation, and bacterial burden following challenge and protection in multiple mouse pneumoniae models (Wang et al., 2016). Protective efficacy of MAb’s 4C5 (IgG1) and 19A10 (IgG3) generated against K1-serotype CPS (K1-CPS) was demonstrated in murine model of sepsis and pulmonary infection. After MAb treatment, reduced dissemination of *K. pneumoniae* from gut to mesenteric lymph nodes and organs, efficient opsonophagocytosis and clearance of bacteria from liver were noted (Diago-Navarro et al., 2017). Apart from these, a humanized MAb (A1102) specific for the conserved LPS O-antigen was found effective for endotoxin neutralization. Passive administration of A1102 showed efficacy more than that of polymyxin B by three orders of magnitude and afforded significant protection in a galactosamine-sensitized mouse model of endotoxemia or upon challenge with live bacteria (Szijártó et al., 2017). Other passive immunotherapy approach to reduce bacterial counts and delayed onset of infection was tried mainly using the antibodies directed against CPSs (Cryz et al., 1985), inactivated whole cells (Cooper and Rowley, 1982), ribosomal preparations (Riottot et al., 1979), cell surface preparations (Fournier et al., 1981), cytotoxins (Singh and Sharma, 2001) and conjugate vaccine preparations (Chhibber and Bajaj, 1995). However, most of these studies were limited to single capsular serotype or single strain. Due to rampant spread of multidrug resistance and the severe nature of *K. pneumoniae* infections, highly protective and specific active immunization and passive immunotherapy approaches should be considered for prophylactic or therapeutic approach for treatment of *K. pneumoniae* infections. Vaccination with OMPs elicits a strong Th17 response against broad-spectrum of *K. pneumoniae* strains along with recently described metallo-beta-lactamase-I producing strains (Chen et al., 2011b). Targeting pathogen-associated molecular pattern molecules (PAMP’s) such as OMPs (OmpA and OmpK36) and antibodies raised against these molecules would form effective bactericidal defense mechanisms against bacterial clearance from the system. Therefore, purified outer membrane preparations could be ideal targets to provide specific and targeted protection against most *K. pneumoniae* strains independent of serotype.

In the present study, immunization with recombinant r-AK36 candidate subunit vaccine comprising highly conserved portions of OmpA and OmpK36 proteins of *K. pneumoniae* conferred protection against intraperitoneal lethal challenge with *K. pneumoniae* E-1716 strain belonging to virulent K2 serotype in murine model. For this model, BALB/c mice that are traditionally used for vaccine studies were utilized despite Th2-skewed immune response. On the other hand, one can speculate that Th2- polarized immune responses render BALB/c mice ineffective to assess cell-mediated immunity. But previous studies in influenza vaccine studies have shown that primary immunization of vaccine induces Th1 immune responses in BALB/c mouse model. Considering these factors we utilized BALB/c mice in this study for vaccination and challenge studies.

Anti-r-AK36 antibodies reacted strongly with all *K. pneumoniae* strains along with many other Gram-negative rods such as *Salmonella* sp., *Shigella* sp., *E. coli*, and *Proteus* sp. indicating a possibility of cross-immunity and cross-protection properties. The antibodies showed significant antimicrobial activity against *K. pneumoniae* strains. Microbial biofilm represents a major virulence mechanism of pathogenic *Klebsiella* sps. associated with chronic and recurrent infections aiding in resistance from chemical and environmental agents (Di Domenico et al., 2016). Anti-r-AK36 antibodies also inhibited the biofilm formation in different *K. pneumoniae* strains belonging to different serotypes. *In vitro* experiments demonstrated that r-AK36 antibodies possessed strong neutralizing and inhibitory properties. Therefore, we presumed that they could offer protection in a *K. pneumoniae* infection model in mice. Passive antibody administration in naive mice conferred partial protection against the lethal challenge. Low viable counts of *K. pneumoniae* were recovered from the vital organs such as lungs, liver, kidney, and spleen of vaccinated animals rather than from the control mice indicating the protective efficacy of the subunit vaccine preparation. Immune response generated by r-AK36 antigen was long lived as seen from the reasonable protection conferred 120 days past last vaccination. The r-AK36 subunit vaccine preparation is more advantageous over reported vaccines due to its protective efficacy, long lived immunity and less or no local anaphylactic reaction at the site of antigen administration. Results obtained from our studies on r-AK36 subunit vaccine were in agreement with some of results obtained by immunizing OmpA and OmpK36 as DNA vaccines in mouse model (Kurupati et al., 2011). Additionally, immunization of *K. pneumoniae*-derived extracellular vesicles (EVs) comprising OMPs also produced similar results as in our studies (Lee et al., 2015). They report that three vaccinations with EV protected against bacterial challenge.

## Conclusion

The data presented in this study identified a novel multicomponent r-AK36 subunit vaccine molecule effective in combating *K. pneumoniae* infectious diseases. This molecule induced both humoral and cell-mediated memory immune responses. This molecule might offer broad-spectrum protection against a majority of *K. pneumoniae* serotypes due to the universal presence of these antigens in all strains irrespective of serotype and their highly conserved nature.

## Author Contributions

LB participated in conceiving, designing, performing the experiments and writing of manuscript. SU helped in writing the manuscript, statistical analysis, revision and proofreading of manuscript. MS helped in work design, planning, providing reagents and other logistics and manuscript evaluation. PR helped in performing experiments, analysis and interpretation of results, writing and critically reviewing the manuscript.

## Conflict of Interest Statement

The authors declare that the research was conducted in the absence of any commercial or financial relationships that could be construed as a potential conflict of interest.

## Abbreviations

CPS: capsular polysaccharide
LD: lethal dose
LPS: lipopolysaccharide
MIC: minimum inhibitory concentration
OMP: outer membrane protein
